# Self-Care Needs and Technology Preferences Among Parents in Marginalized Communities: Participatory Design Study

**DOI:** 10.2196/27542

**Published:** 2021-06-22

**Authors:** Weichao Yuwen, Miriana Duran, Minghui Tan, Teresa M Ward, Sunny Chieh Cheng, Magaly Ramirez

**Affiliations:** 1 School of Nursing & Healthcare Leadership University of Washington Tacoma Tacoma, WA United States; 2 Department of Health Services School of Public Health University of Washington Seattle, WA United States; 3 School of Nursing University of Washington Seattle, WA United States

**Keywords:** asthma, caregiving, self-care, parents, qualitative research, culturally appropriate technology, minority groups, marginalization

## Abstract

**Background:**

Ten million parents provide unpaid care to children living with chronic conditions, such as asthma, and a high percentage of these parents are in marginalized communities, including racial and ethnic minority and low-income families. There is an urgent need to develop technology-enabled tailored solutions to support the self-care needs of these parents.

**Objective:**

This study aimed to use a participatory design approach to describe and compare Latino and non-Latino parents’ current self-care practices, needs, and technology preferences when caring for children with asthma in marginalized communities.

**Methods:**

The participatory design approach was used to actively engage intended users in the design process and empower them to identify needs and generate design ideas to meet those needs.

**Results:**

Thirteen stakeholders participated in three design sessions. We described Latino and non-Latino parents’ similarities in self-care practices and cultural-specific preferences. When coming up with ideas of technologies for self-care, non-Latino parents focused on improving caregiving stress through journaling, daily affirmations, and tracking feelings, while Latino parents focused more on relaxation and entertainment.

**Conclusions:**

Considerations need to be taken beyond language differences when developing technology-enabled interventions for diverse populations. The community partnership approach strengthened the study’s inclusive design.

## Introduction

One in four children in the United States has chronic health conditions, such as asthma and diabetes, and those who reside in marginalized communities, including racial and ethnic minority and low-income families, are disproportionately affected [1-4]. An estimated 10 million parents provide unpaid care to children living with chronic conditions, and a high percentage of these parents are part of marginalized communities. The responsibilities and demands of parents of these children are 24/7 and multifaceted (eg, monitor symptoms, administer medications or therapies, and nighttime care). Over time, these responsibilities and demands take a toll on the physical and mental health and work of parents and family relationships, and contribute to the symptoms of fatigue, distress, disturbed sleep, and anxiety [5]. These symptoms are negatively associated with parent well-being (quality of life and family and social function), which impacts the health outcomes of children [6]. Self-care is defined as “the ability of individuals, families, and communities to promote health, prevent disease, maintain health, and cope with illness and disability with or without the support of a health care provider” [7]. Despite the pervasiveness of these symptoms, parents often receive no training in caring for their own health. Existing interventions to improve parent symptoms of distress, fatigue, anxiety, and sleep have primarily focused on caregivers of adult populations with dementia or parents of pediatric cancer populations with moderate to high socioeconomic status [8,9]. Less is known about interventions that focus on self-care for parents residing in marginalized communities [10]. The COVID-19 pandemic has worsened the existing caregiving crisis. Effective digital health strategies can support these families virtually during the pandemic and remain useful after the pandemic [11].

In the United States, parents of children with chronic health conditions are more likely to be racial and ethnic minorities than caregivers of adults, with Latinos representing the largest minority group [12]. Nonetheless, Latinos and parents from other historically marginalized groups are underrepresented in studies of interventions that aim to support parents of children with chronic conditions [5,13]. Most published studies that explore the use of technology to enable the delivery of supportive interventions for parents of children with chronic conditions were from Europe [14-18]. To our knowledge, only one published study has been conducted in the United States, and it focused on the technology preferences of parents of children with shunted hydrocephalus, but the sample only included 1% Latinos [14]. Currently, in the United States, Latinos represent one of the largest racial/ethnic groups who care for children with chronic conditions, and their representation in parent research is lacking. Lack of representation from marginalized communities, including racial and ethnic minority and low-income families, in designing technology-enabled interventions can increase the risk of intervention-generated inequalities, including further decreased access, adoption, and updates; poor adherence; and reduced effectiveness, for people in marginalized communities [19].

Perspectives from these parents are needed to design inclusive technology-enabled interventions that support self-care among diverse populations of parents caring for children with asthma. Otherwise, design decisions can inadvertently result in intervention-generated inequalities [19]. The types of technology devices used to deliver interventions and the delivery settings (eg, workplace wellness programs and health care organizations) may be less accessible to marginalized groups [20-22]. Even within groups with equal access to technology-enabled interventions, more significant attrition and lower adherence rates were observed in groups with lower educational attainment levels [23-25]. These inequalities could be attributed to the lack of representation in the intervention design and development phases, leading to inappropriate cultural, linguistic, literacy-level, and economic-level intervention treatment components, delivery, and engagement [19].

Participatory design is a promising approach to designing inclusive parent interventions that minimize design decisions inadvertently causing intervention-generated inequalities. The premise of participatory design is to actively engage intended users in the design process and empower them to identify needs and generate design ideas to meet those needs [26]. Based on the information technology literature [27], we expect that utilizing participatory design to design technology-enabled interventions would result in a better understanding of parents’ needs and design requirements, a greater likelihood of including features that parents want, a higher level of acceptance, and an improved understanding of how the technology-enabled intervention works. As Clemensen et al described, participatory design is an iterative process that involves the following: (1) engaging intended users in identifying needs, (2) empowering intended users to generate ideas for solutions that could address these needs, (3) pilot testing and refining these solutions, and (4) assessing the effectiveness of the solutions [28]. We adopted a participatory design approach to design an inclusive technology-based intervention that promotes self-care among predominantly low-income and Latino parents of children with asthma. In this paper, we describe the first two steps in the participatory design process.

The purpose of this study was to use a participatory design approach to describe and compare Latino and non-Latino parents’ current self-care practices and needs, and to identify how to leverage technology to enhance self-care practices among parents of children with asthma.

## Methods

### Design

This descriptive qualitative study is part of a larger study in designing and developing a technology-enabled mobile health intervention for parents of children with chronic conditions (Caring of Caregivers Online [COCO]) [29].

### Sample and Sampling

We recruited stakeholders (parents and clinical experts) from an asthma coalition established with multiorganizational effort to improve the quality of life for individuals, families, and communities affected by asthma in the northwestern United States. The coalition is a community, clinical, and academic partnership to strengthen relationships among stakeholders (eg, patients, families, clinicians, researchers, and community health workers), particularly in marginalized communities underrepresented in research. There are two parent advisory groups in the coalition, one Latino and one non-Latino group. The first author is a long-term member of the coalition who presented CocoBot at multiple coalition monthly meetings to receive input on study conceptualization, grant preparation, study design, and recruitment. The research team shared the study recruitment flyer for this study with the coalition members, who distributed the flyers in the community, including child care centers, asthma clinics, and community health centers. The inclusion criteria for parents were as follows: (1) age 18 years or older; (2) ability to read and speak English or Spanish; (3) having a child (aged 3 to 12 years) with asthma; and (4) co-residing with the affected child more than 50% of the time. A parent was defined as the child’s primary caregiver (biological or adopted). In addition, we invited members of the coalition who were not parents of children with asthma to participate in a clinical expert participatory design session. The inclusion criteria for clinical experts were as follows: (1) age 18 years or older; (2) ability to read and speak English; and (3) working with families of children with asthma, including health care professionals working at different settings caring for these children and their families, local health department staff, and community health workers. Individuals who were interested in study participation contacted a study team member who screened them for eligibility. All study participants provided written informed consent. All participants who met the inclusion criteria were enrolled and completed the study.

### Procedures

We conducted three in-person participatory design sessions and mailed participatory design packets for additional input. Three participatory design sessions were conducted with five Spanish-speaking parents (all Latino), four English-speaking parents (one Latino and three non-Latino), and three clinical experts in childhood asthma (one Latino and two non-Latino) in a private conference room at a public library or the coalition regular meeting space. Participatory design sessions lasted 55 to 80 minutes. We obtained audio and video recordings of the sessions and collected all artifacts generated by participants. We later invited study participants to a follow-up with written responses to elaborate on the topics discussed during the participatory design sessions. Two Latino parents, who were interested in participating but were not available to attend an in-person participatory design session, were invited to provide written responses to topics discussed during the participatory design sessions with a mailed participatory design packet. The packet contained a consent form, a demographic questionnaire, ideation materials (pens, markers, drawing paper, and post-it notes), and forms that guided study participants through the ideation process. A team member scheduled a phone session with the participants to explain the packet and questions, and instructed the participants to return the packets with completed responses via the US Postal Service. We received one packet back. The other parent said the packet could not be found after 2 months and did not want to receive another packet. The institutional review board approved all study procedures.

### Data Collection

The participatory design session was designed to actively engage study participants in interactive discussions to co-design potential technology solutions to support the self-care of parents’ symptoms. In the first part of the participatory design session, we asked study participants to write down parents’ most common caregiving concerns and symptoms, self-care practices, and needs. We asked study participants to share their ideas with the larger group. The facilitator then used affinity diagramming to organize ideas into groupings based on common themes. In the second part of the participatory design session, we provided study participants with ideation materials (pens, markers, drawing paper, and post-it notes). We asked them to work individually to sketch ideas for future technology solutions that could address parents’ caregiving symptoms and self-care needs. We asked participants to visually share their ideas with the larger group and encouraged other study participants to ask questions and provide constructive feedback. We ended the participatory design session by asking participants to share additional technology features they could envision incorporating into parents’ daily lives for self-care.

### Data Analysis

**Figure 1:**
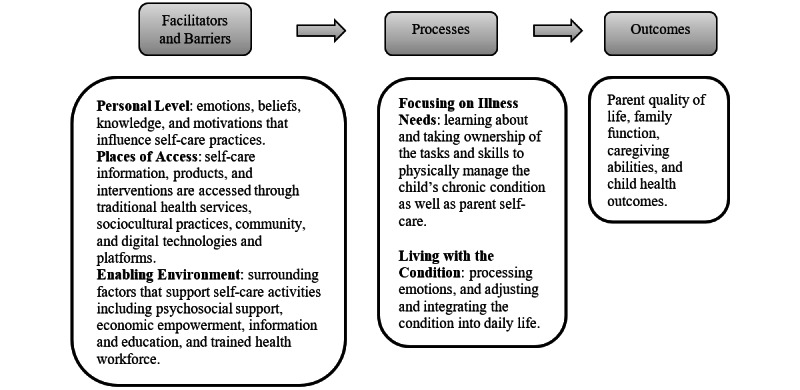
Conceptual framework for self-care among parents caring for children with chronic conditions.

To guide data analysis, we developed a conceptual framework (Figure 1) based on the Self- and Family Management Framework [30] and World Health Organization’s (WHO’s) Conceptual Framework for Self-Care [31]. The Self- and Family Management Framework describes facilitators and barriers to, processes involved in, and outcomes of self-management and family management of chronic conditions [30]. The facilitators and barriers to family management focus on traditional practices such as personal factors, the environment, and health care systems. WHO’s Conceptual Framework for Self-Care complements the Self- and Family Management Framework by expanding the places of access and enabling the environment for self-care, including traditional routes and emerging digital health and technologies [31]. The latter is a salient field of practice to expand access to health information and communication, address health needs, and reduce health inequalities [31].

Members of the research team transcribed audio recordings verbatim, transcribed participatory design session artifacts, and transcribed participatory design packet responses in English or Spanish. Two members of the research team with qualitative research experience deductively coded the unitized data (English and Spanish) independently and used the concepts as themes according to the conceptual framework [32]. Because we focused on self-care practices and needs, we did not present the conceptual framework’s outcomes in this study. Transcripts were coded in their original language. A codebook was developed with definitions and representative quotes for each domain and category. Two additional research team members performed peer debriefing on the coding in English and Spanish, according to the codebook. We used constant comparative analysis to examine each unit’s fit with the coded domain and category, and the uniqueness of each unit’s coding (no unit fit under two different categories) [33]. Disagreements were discussed among all four team members in a series of meetings, resulting in recoding a unit or refinement of the codebook until 100% consensus.

## Results

### Demographic Characteristics

Table 1 shows the demographic characteristics of Latino and non-Latino stakeholders. We combined the parents and clinical experts into the two stakeholder groups by ethnicity to protect participant confidentiality. The majority of parents were female (9/10, 90%), without a college degree (8/10, 80%), and employed outside the home (6/10, 60%). Most non-Latino parents earned less than US $19,000 annual household income (*n* not reported for confidentiality). The majority (4/7, 57%) of Latino parents earned less than US $60,000. All parents self-identified belonging to at least one of the following two marginalized communities of focus in this study: racial and ethnic minority and low-income families.

**Table 1:** Characteristics of non-Latino and Latino stakeholders (parents and clinical experts).

Characteristic		Non-Latino stakeholders (n=5)	Latino stakeholders (n=8)
Age (years), mean (SD)		48 (11.5)	42 (7.1)
Female, n (%)		4 (80%)	8 (100%)
**Race/ethnicity, n (%)**			
	White	3 (60%)	0 (0%)
	Black	2 (40%)	0 (0%)
	Latino	0 (0%)	8 (100%)
**Education, n (%)**			
	Less than high school	0 (0%)	1 (12.5%)
	High school	1 (20%)	5 (62.5%)
	Some college, no degree	2 (40%)	0 (0%)
	Associate or bachelor’s degree	2 (40%)	2 (25%)
**Employment status**			
	Working now	5 (100%)	3 (37.5%)
	Self-employed	0 (0%)	3 (37.5%)
	Homemaker	0 (0%)	1 (12.5%)
**Marital status**			
	Married	2 (40%)	6 (75%)
	Single (eg, divorced and widow)	3 (60%)	2 (25%)

In the following sections, we present the barriers and facilitators to self-care related to personal factors, places of access, and the environment. We also present processes for self-care, including focusing on illness needs and living with the condition. In each section, we highlight the technology preferences among the two groups.

### Facilitators and Barriers

#### Personal Level

At the personal level, non-Latino parents experienced various emotions caring for their children with asthma, including worry, stress, fear, guilt, frustration, and isolation. Parents were unaware of supportive services that were available to help them manage these emotions. An example response was as follows:

Depression? There are no easy resources... I never had one of my children’s healthcare providers ever asked or checked in.

Non-Latino parents with multiple children with asthma expressed that it was challenging to fully understand each child’s care needs because asthma affected each child differently (eg, children within the same family had different asthma triggers). Moreover, while non-Latino parents perceived a connection between the well-being of the parent and that of the child with asthma, they nonetheless found it difficult to justify taking the time to care for their own health (Table 2).

Similar to non-Latino parents, Latino parents expressed that being a parent of a child with asthma led to them experiencing various emotions, including fear, worry, stress, guilt, hopelessness, and frustration. Latino parents felt these emotions when they thought about whether the precautions they were taking to reduce asthma triggers in their home would be useful, how to respond in an emergency, and how they had little to no control of the environment outside of the home (eg, school) that could trigger an asthma attack. Latino parents described prioritizing their children’s needs before their own as part of the Latino culture. On discussing their self-care, Latino parents indicated that they could not focus on their self-care until the rest of their family members were taken care of. For example, one clinical expert made the following statement:

For my community, we’ve always taken pride in taking care of our family before we take care of ourselves.

When asked to generate ideas for how technology could address personal barriers to self-care, non-Latino parents mentioned reminders for self-care activities, including breathing and mindfulness exercises, relaxation, healthy recipes, and tracking feelings. Latino parents suggested that the information presented on digital devices be clear and concise, and minimize distractions. They also wanted the information to be accessible in English and Spanish, and designed with both parents and children as the intended audience. Finally, Latino parents described their desire to have a technology tool that presents information for both parents and children to learn about self-care in an informative, fun, and entertaining way.

**Table 2:** Descriptions and representative quotes from non-Latino and Latino stakeholders (parents and clinical experts).

Domain and category			Quotes from non-Latino stakeholders		Quotes from Latino stakeholders
**Domain 1: Facilitators and barriers**					
	Personal level	*The child has asthma and it’s hard to separate and it’s impossible to separate when it’s about caring for them or when it’s caring for us, right? Or if they get better, we’ll get better too, so it’s kind of hard to say um… I’m going to do this just to care for myself or do this just to care for my child.* *[Parents] don’t realize how the lack of sleep, mood or how that might affect their health and wellbeing I don’t think [parents] recognize for themselves or they see it right or they allow themselves to have those perks.*		*Como me siento, pues me siento asustada, estresada, preocupada. Me asusta el saber, el no saber si las medidas que estoy haciendo que estoy tomando para mis hijos funcionan. Si en caso de emergencia voy a saber qué hacer y si sé que hacer,entonces, si va a funcionar para mi hogar.* Translation: *How I feel, well I feel scared, stressed, worried. It scares me knowing, not knowing if the measures that I am doing that I am taking for my children work. If an emergency happens, will I know what to do and if I know what to do, then if it will work for my child.*	
	Places of access	*Other family members who might help or not help them [parents] care for their child and the siblings.* *B* *aby monitor in his [child’s] room where I can hear and if I hear anything that doesn’t sound right I can like know, I can go back.*		*Bueno, a mi lo que me funciona cuando mi niño está enfermo y me siento frustrada y eso, es hablar con alguien, hablar con una amiga. Eso me relaja mucho.* Translation: *Well, what works for me when my child is sick and I feel frustrated is to talk to someone, to talk to a friend. That helps me a lot to relax.*	
	Enabling environment	*The doctors...don’t understand that the way I’m caring for my children and the events going on at home you know you’re not seeing what I’m seeing but I’m seeing it and they [doctors] will say like, you know, um [acting as the doctor not believing], just getting frustrated because you feel helpless.* *[I was] struggling to get school staff to take the illness seriously and administer the medicine responsibly and appropriately.*		*Pienso que a veces los doctores tienen muy poquito tiempo para nosotros, pero pienso que a veces pienso que ni siquiera ellos no tienen toda la información tampoco.* Translation: *I think that sometimes doctors have very little time for us, but I also think that sometimes, not even they have all the* *information.* *Que tenga un ataque de asma en la escuela y la enfermera no sepa que hacer que en un ataque de asma y mi hijo deje de respirar.* Translation: *[Concerns as a parent]* *that my child has an asthma attack at school and the nurse does not know what to do when a child is having an asthma attack and my child stops breathing.”*	
**Domain 2: Processes**					
	Focusing on illness needs	*You [parent] have a child who you want to self-carry their rescue inhaler.* *You [parent] kind of lose control a little bit of what they [the child] do when they’re older.*		*Que ellos tengan conocimiento de que, si tienen una condición que sepan que tienen que hacer, eso les da a ellos seguridad en sí mismos, ellos estar informados y estar envueltos en su propia condición, en su tratamiento, eso ayuda para que ellos no tengan miedo de hacer cosas, de jugar, de correr, de hacer ejercicio.* Translation: *They (children) need to understand that if they have a health condition, they need to know what to do. This will help them to build self-confidence. They need to be informed about and involved in their own condition and its treatment. This will help them to avoid feeling afraid of doing things, such as playing, running, and exercising.*	
	Living with the condition	*How am I going to go to the gym if my kid is sick at home, how am I going to eat this if it’s really good if my kid can’t have it, how am I going to go out on vacation if my kid I need to afford this medication, who is going to take care of them the same way I do?* *I just need to tough it out, right? Like, I don’t care about myself like, I just need to pull it through to care for my child.* *Complaining to each other.* *Trying to do [everything] everyday - it’s always like, is this enough?*		*Mientras ellos [hijos] están pasando por una cosa así, no puedo tener mucho con que sentirme bien. No creo que mientras que ellos estén mal, yo pueda relajarme o cosas así. Entonces lo que pasa es, por ejemplo, me ayuda el saber la reasuransa o “reassurance” de que van a estar bien... Y si puedo tener eso, entonces ya puedo tener las otras cosas [autocuidado].* Translation: *If my child is going through something (asthma symptoms), there is not a lot that will help me feel better. While they are not doing well, I don’t think that I could relax or anything like that. So what happens, for example, is that it helps me when I can be reassured that they are going to be okay… once I have that reassurance, then I can have these other things [related to my self-care].*	
	Technology preferences	*Voice would be better than writing it down.* *Seasonal reminder related to asthma.* *There are games on the phone and people can collect different like if you [parent] answer these four question you get like an award and then* *s* *ee.* *..and I don’t know if other people would like that, it’s not for me, but I know other people do those things that they get excited about, they have evidence of what they have done.* *Reminders to take time to breathe* *[for the parents]* *.* *Help you [parent] with reminders of journaling, daily affirmations and could ask you how you feel.* *They [parents] liked getting the text message weekly because that didn’t feel overwhelming.*		*Una buena idea sería que haya videos de mamas con hijos con asma y saber su experiencia, entrevistas en TV o en radio, también entrevistas en persona y mostrar fotos de los lugares en casa no recomendables para niños con asma. También fotos de los productos de limpieza que usan en el hogar.* Translation: *A good idea would be to have videos of moms with their children with asthma, to learn about their experience, on TV or on the radio. Also, interviews that show photos of places in the home that are not recommended for children with asthma. Also, photos of cleaning products that are safe to use in the home.* *Uso cel para oir musica para relajarme.* Translation: *I use my cellphone to listen to music and relax.* *Uso mi celular para buscar informacion.* Translation: *I use my cellphone to look for information.*	

#### Places of Access

There were barriers and facilitators regarding the places that parents accessed self-care support. In their communities, non-Latino parents turned to family members and friends to care for their children with asthma and other siblings while the parents took short breaks from caregiving. Although non-Latino parents trusted their family members and friends, they still felt apprehensive about leaving their children with asthma under the care of another person. Non-Latino parents were worried about their children experiencing an asthma attack when they are away. They explained how the family members or friends caring for their children might not know what would trigger an asthma attack or how to respond if the children experienced one. One non-Latino parent made the following statement:

You [the parent] trust the person, but something can go wrong, and it could be a small thing that they don’t notice, or anything could lead to a big asthma attack.

Latino parents also relied on family members and friends for self-care support, but in a different form than non-Latino parents. For Latino parents, family members and friends were a source of emotional rather than tangible support. For example, one Latino parent mentioned that speaking with a friend was an effective strategy for easing the difficult emotions associated with caring for a child with asthma (see Table 2 [quotes from Latino stakeholders]). Unlike non-Latino parents, sociocultural practices were a place of access for self-care support among Latino parents. In particular, Latino parents described the vital role of religion in their ability to cope with the challenges of having a child with asthma.

Mobile technologies were also points of access for self-care support for both groups of parents. Non-Latino parents described using apps for self-guided meditation, medication reminders, asthma management, and parenting advice. Parents used baby monitors in their children’s bedrooms to hear when they struggled with asthma symptoms at night. Similarly, Latino parents described searching for information on the internet to learn about asthma and its management. Latino parents reported using apps to listen to music and watch shows and movies to help manage their stress. They also used technology to create reminders of upcoming doctor appointments, get recipes for healthy meals, share experiences with others through social media, and track physical activity. While Latino parents recognized the advantages of mobile technologies for accessing self-care support, there was concern about how easy it was to get distracted by and addicted to technology. To that end, Latino parents desired to obtain more of the information from other sources, including asthma-related community events, health care providers, television, and radio. One Latina mother shared ideas for how information could be disseminated to parents of children with asthma using different media forms (Table 2).

#### Enabling Environment

There were barriers and facilitators related to the environments in which parents accessed self-care support. While non-Latino parents used mobile devices to access information on both parent and child self-care, parents felt overwhelmed by the vast amount of information they consumed on the topic. Non-Latino parents also discussed feeling anxious when searching for information online, and they encountered stories about asthma-related accidents experienced by children. While health professionals were generally helpful, non-Latino parents reported that sometimes different health professionals provided conflicting information. One parent made the following statement:

Every doctor is different so they sometimes give different recommendations and you do what they tell you and then you do another one there

as another doctor tells you another recommendation

In addition, non-Latino parents were dissatisfied with what they perceived to be health professionals’ lack of knowledge about their family and parenting style. Health professionals were unable to provide information tailored to the unique situation of their family. One parent made the following statement:

They [the doctors] are not listening, or maybe part of it is not listening, and part of it is not respecting the parent’s wisdom… their wishes and wisdom about their child’s asthma.

Like non-Latino parents, Latino parents used various mobile devices (mostly smartphones) to access self- and child-care information. Latino parents discussed other environments in which they accessed self-care support, including asthma-related meetings and community events. While some Latino parents were English speakers, it was still of considerable importance that high-quality information about asthma and self-care for both parents and children be accessible in Spanish. Furthermore, Latino parents discussed accessing self-care support from health professionals. A theme among Latino parents, which was not observed among non-Latino parents, was that the former group said medical doctors did not provide sufficient education and support during clinic visits. One Latino parent described her desire for doctors to educate families on what changes they could make to have an asthma-safe home for their children. This parent explained possible reasons for the lack of education and support for families on the behalf of doctors (Table 2).

In addition to medical doctors, both non-Latino and Latino parents identified the school as a potential environment to access asthma support. However, both groups of parents generally lacked confidence in school nurses’ ability to respond to their children’s asthma attacks effectively. One Latino parent described her concerns about having school nurses without proper education and asthma care training (Table 2).

When providing education to parents, both groups pointed out that health professionals need to present information in small chunks over time, rather than “dumping information all at once.” Breaking down information into bite-sized pieces would make it easier for parents to gradually implement changes in their home environment, rather than trying to change everything at once, as one non-Latino parent suggested. Regarding Latino parents with children with asthma, one clinical expert made the following statement:

They are so concerned about doing everything perfect that it takes all their time. It consumes their life trying to get everything perfect. Then, they’re not taking care of themselves or doing anything else, you know?

### Processes for Self-Care

#### Focusing on Illness Needs

Learning about asthma management and self-care is long term and complex. Non-Latino parents shared that they had to constantly struggle to let go of the idea that asthma is a serious condition in order to focus on management strategies one by one. There was a recognition in both groups that developing effective asthma management skills must happen slowly over time. All parents were proactive in learning how to create an asthma-safe home for their children, for example, “products [that] are triggers for our kids, so cleaners, clothes, and scents.” Furthermore, parents in both groups discussed encouraging older children with asthma to take greater ownership of their asthma management. One Latino parent described why it was important to transfer asthma management responsibility from the parent to the child (Table 2). However, older children sometimes refused to use their inhaler because they perceived it as no longer needed. For this reason, non-Latino parents felt reluctant to transition self-care into a shared responsibility between parents and older children with asthma (Table 2).

#### Living With the Condition

Parents discussed how they coped with their children’s asthma and integrated the condition into daily life. Both groups of parents felt that they could not focus on their self-care until they knew their children were well. To provide the best care to their children, non-Latino parents believed it was necessary to process the difficult emotions associated with their children having asthma. Strategies that non-Latino parents found to help them cope with the condition included talking to family members and friends, journaling, meditating, reading affirmations, and setting self-care goals. Some non-Latino parents shared that working outside of the home provided temporary relief from these difficult emotions. Their work required them to be fully engaged instead of thinking about their children’s health condition. However, non-Latino parents reported having to miss a significant amount of work because of their children’s asthma symptoms.

Latino parents were reluctant to consult a mental health professional for their self-care support. One Latino clinical expert made the following statement:

We [Latinos] don’t do medication, we don’t do a lot of stuff that you people don’t even see, you know? I don’t need to go to a psychologist when I’m not crazy.

Instead, Latino parents described different ways they coped with their own difficult emotions. Strategies included listening to music, going outdoors for a walk, increasing their knowledge about asthma, and working with their children to manage asthma better. Latino parents described adjustments they have made in their homes and lifestyles to manage their children’s asthma. Namely, Latino parents switched to cleaning with non-toxic products, began cooking healthier meals, and incorporated more daily physical activity for the entire family. Similar to non-Latino parents, it was not until Latino parents observed improvements in their children’s asthma symptoms that they felt they could focus on their self-care (Table 2). One Latina mother, who considered herself a Catholic, described putting her trust in God. While she would continue to do what was necessary and under her control to manage her child’s asthma, it helped to accept that what ultimately happens is the will of God.

### Co-Designed Solutions to Address Self-Care

**Figure 2:**
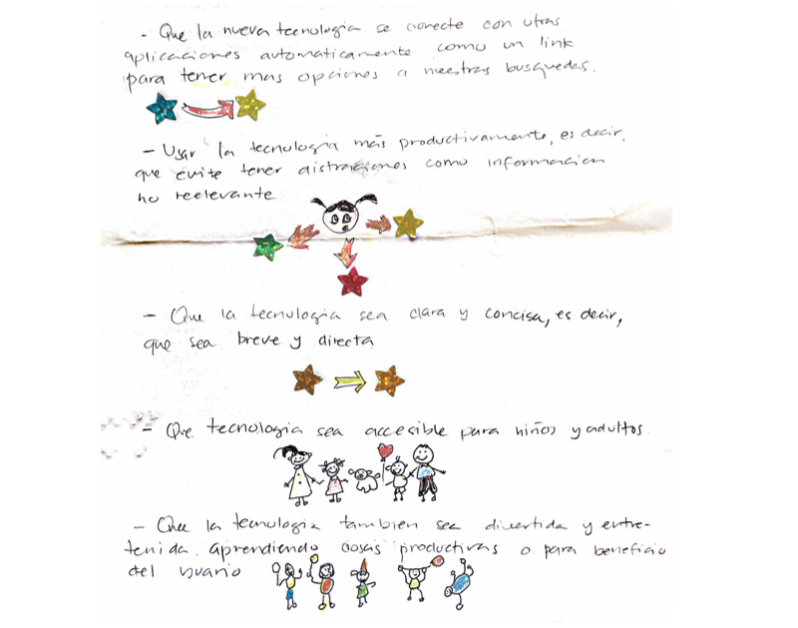
Example design artifact from a Latino parent. Technology ideas for self-care: new technology connects with other apps automatically to have more search options; use technology productively, without distractions or irrelevant information; have clear and concise information; technology should be accessible for children and adults; and technology should be fun and entertaining.

**Figure 3:**
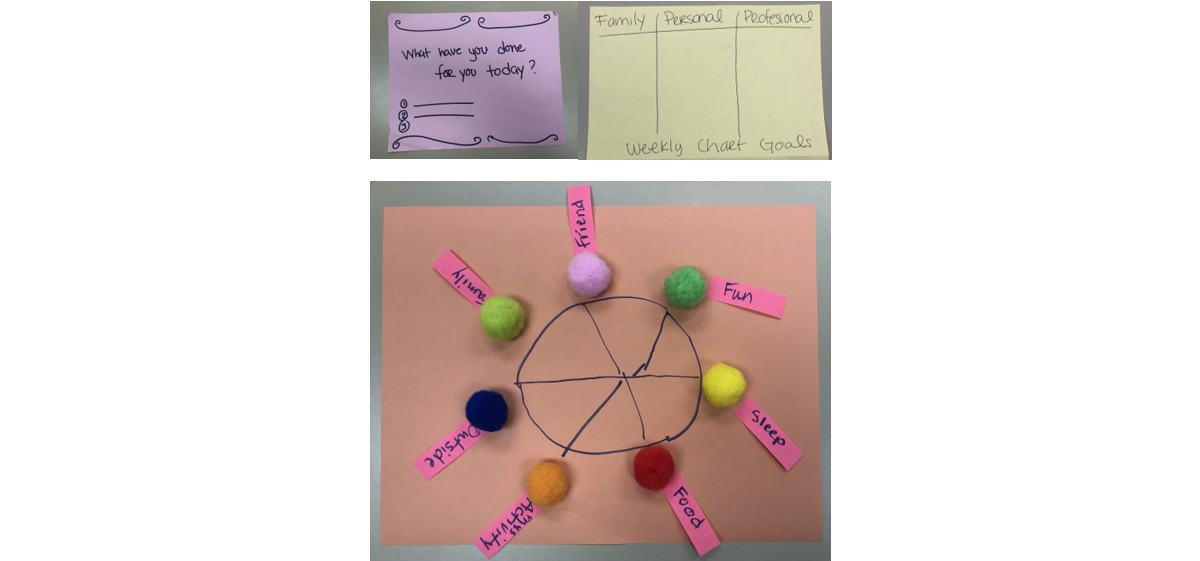
Example design artifacts from non-Latino parents. From left to right, top to bottom: reminder for self-care, setting weekly goals, and pie chart for self-care activities.

Study participants co-designed potential technology solutions to support both parent and child self-care needs (see Figures 2 and 3 for examples from Latino and non-Latino parents, respectively, and Table 2 for quotes). Both groups of parents described technology features that would enable their children with asthma to use technology to set reminders to take their medications. Both groups of parents also described wanting to be reminded to take deep breaths and find recipes for healthy meals. Non-Latino parents further described technology features that would enable them to share an asthma care plan with others (eg, school nurses and other parents), receive affirmations, practice journaling, track their feelings, and track information about asthma attacks (eg, when and where they happened) to identify patterns. Latino parents described technology features that would enable them to search for information about asthma and its management, set reminders for upcoming doctor appointments, listen to music, watch shows and movies, watch videos of other mothers discussing their experiences, find cleaning products that are safe to use in the home, track tasks, and track physical activity.

## Discussion

### Principal Findings

This study provided new knowledge about self-care needs and technology preferences among Latino and non-Latino parents in marginalized communities. Both groups of parents experienced negative emotions that would hinder their parenting abilities but could not prioritize taking care of themselves over caring for their children. The two groups differed in the places of access for self-care support, such as families, friends, and communities. Both groups of parents accessed caregiving information online and through health care providers. However, the information was perceived as overwhelming and not tailored to their specific needs. Current self-care practices using technology were similar among the two groups, including using applications for relaxation. When coming up with ideas of technologies for self-care, non-Latino parents focused on improving caregiving stress through journaling, daily affirmations, and tracking feelings, while Latino parents focused more on relaxation and entertainment.

### Comparison With Prior Work

Our study found that both Latino and non-Latino parents were interested in the idea of using technology to access support for their self-care. Parents generated ideas for future technology tools that incorporated support for both the physical and mental health aspects of self-care, such as learning to make healthier meals and practicing deep breathing for relaxation. Research on the technology needs and preferences of parents caring for children with chronic health conditions is limited, particularly among historically marginalized populations. To our knowledge, we found only one published study on the topic, which focused on understanding parents’ current use of technology devices and their online sources of information [14]. Notably, non-English speakers were excluded from the study, and the majority of participants were White. Nonetheless, our findings are complementary. Our study reported that parents used multiple technology devices in their everyday lives and identified the internet as a significant source of information. Further, in our research, we found that Latino parents’ ideas for future technology tools included the requirement that information is not only educational but also entertaining. There is evidence to suggest that entertainment education can be an effective strategy to improve access to health-related information among Latino populations [34-36], though additional research is needed to understand the potential impact of delivering entertainment education via digital tools. Finally, our findings showed the importance of developing technology tools that clearly and concisely present information in both English and Spanish. This finding highlights the unmet need for disseminating culturally and linguistically appropriate technology-enabled interventions among Spanish-speaking Latino populations [37].

### Limitations

This research took place as part of an academic-community partnership between the University of Washington researchers and the Puget Sound Asthma Coalition and their multicultural parent advisory groups. Although partnering with a community-based organization strengthened the study’s inclusive design, the results were limited as most of the participants were already members of the coalition. Thus, they were more knowledgeable about the research process and had experiences engaging in research in the past. The clinical experts also had personal experiences and connections with asthma; thus, we cannot rule out that some parents may have shared experiences as parents of children with asthma, along with their clinical experiences. One challenge in conducting this community-based bilingual research was that some participants belonged to multiple sociocultural groups (eg, Latino and preference for speaking English). We organized the participatory design sessions by preferred spoken language but analyzed the data by ethnicity. Other participants could have influenced Latino stakeholders in the English-speaking group. We did try to overcome this by asking participants to respond to questions and design prompts individually first and then share the responses with the larger group. We had a relatively small sample size as the data collection had to be stopped due to the start of the COVID-19 pandemic. The research team analyzed the data received and determined that we had sufficient data to conduct analyses. In addition, we were only able to conduct the first two steps of participatory design in this study. The research team is actively pursuing additional funding to continue this project.

### Conclusions

This study is one of the first to use the first two steps of participatory design to engage stakeholders in marginalized communities to design a technology-enabled solution for family caregivers to engage in self-care. The community partnership approach strengthened the study’s inclusive design. The COVID-19 pandemic has substantially increased interest in and acceptance of supportive services delivered entirely in virtual environments. In our research using participatory design to engage Latino and non-Latino parents from marginalized communities in identifying needs and generating ideas for solutions that could address these needs, we found that parents in both groups had some similarities in self-care practices but that there were also culture-specific preferences. Considerations need to be taken beyond language differences when developing technology-enabled interventions for diverse populations.

## Abbreviations

WHO: World Health Organization
